# Hexanoic Acid Improves Metabolic Health in Mice Fed High-Fat Diet

**DOI:** 10.3390/nu17172868

**Published:** 2025-09-04

**Authors:** Takako Ikeda, Kumika Takii, Yuna Omichi, Yuki Nishimoto, Daisuke Ichikawa, Tomoka Matsunaga, Ami Kawauchi, Ikuo Kimura

**Affiliations:** 1 Laboratory of Molecular Neurobiology, Graduate School of Biostudies, Kyoto University, Sakyo-ku, Kyoto 606-8501, Japankimura.ikuo.7x@kyoto-u.ac.jp (I.K.); 2 Department of Molecular Endocrinology, Graduate School of Pharmaceutical Sciences, Kyoto University, Sakyo-ku, Kyoto 606-8501, Japan

**Keywords:** hexanoic acid, lipid metabolism, glucose metabolism, insulin resistance, obesity

## Abstract

**Background:** Overweight and obesity are currently a worldwide problem, with undesirable health consequences, such as type 2 diabetes. Therefore, much attention has been paid to preventing obesity through diet. Free fatty acids (FFAs) serve as signaling molecules in many biological processes, leading to increased energy expenditure and insulin secretion. Short-chain fatty acids (SCFAs) such as acetic, propionic and butyric acid are bioactive metabolites produced by gut microbes, and their beneficial effects on host metabolism are well studied. However, the effects of hexanoic acid on metabolism are poorly understood. **Methods:** Male C57BL/6J mice were fed a normal chow diet, a high-fat diet (HFD), an HFD containing 5% butyric acid or an HFD containing 5% hexanoic acid for 4 weeks, and the effects of hexanoic acid on their lipid and glucose metabolisms were examined. **Results:** Dietary supplementation of hexanoic acid or butyric acid for 4 weeks prevented HFD-induced obesity and fat accumulation in the white adipose tissues. Both FFAs also suppressed the elevated plasma non-esterified fatty acid (NEFA) levels and hepatic triglyceride content in the mice fed an HFD. In addition, butyric acid and hexanoic acid decreased the elevated expression of genes involved in fatty acid biosynthesis in the white adipose tissues under HFD conditions. Hyperinsulinemia induced by HFD feeding was attenuated by oral intake of butyric acid or hexanoic acid, whereas hyperglycemia under HFD feeding was improved only through oral administration of hexanoic acid. Hexanoic acid increased plasma glucagon-like peptide-1 (GLP-1) levels and the expression of genes associated with gluconeogenesis. The intraperitoneal glucose tolerance test (IPGTT) and the insulin tolerance test (ITT) revealed that the oral administration of hexanoic acid significantly enhanced glucose tolerance and insulin sensitivity. **Conclusions:** This study highlights the importance of hexanoic acid in improving lipid and glucose metabolisms. Hexanoic acid, as well as butyric acid, is a remarkable FFA with anti-obesity properties. Furthermore, hexanoic acid is more potent in maintaining glucose homeostasis than butyric acid. Thus, our findings provide insight into the development of functional foods which could prevent obesity-related diseases such as type 2 diabetes.

## 1. Introduction

It is currently estimated that more than a billion people are obese, and thus, obesity is a major public health problem worldwide [1]. Obesity is defined by the excess deposition of fat in the white adipose tissues, which modulates lipid and glucose metabolisms by releasing NEFA and hormones [2]. In obese patients, increased plasma levels of NEFA are observed and prevent insulin signaling. In contrast, the secretion of the adipocyte-derived hormone adiponectin is decreased in obesity, and plasma adiponectin levels are negatively correlated with body mass index (BMI) [2]. Adiponectin enhances insulin sensitivity by increasing fatty acid oxidation. Hence, obesity is a major risk factor for type 2 diabetes by causing insulin resistance. In addition, overweight and obesity are associated with an increased risk of cardiovascular diseases [3]. Hyperlipidemia causes atherosclerotic plaque formation in the aorta and coronary arteries, which leads to an increased risk of heart attacks and stroke. Endothelial cell dysfunction in obesity is caused by an increase in the production of reactive oxygen species (ROS), and some natural compounds such as *Withania somnifera* enhance vascular endothelial function by reducing ROS levels [3]. Therefore, there is a growing need for the development and availability of functional foods with anti-obesity properties.

As one of the most effective functional food ingredients, free fatty acids (FFAs) have gained attention. FFAs are classified into the following three groups depending on their carbon chain length: short-chain fatty acids (SCFAs; fewer than 6 carbons), medium-chain fatty acids (MCFAs; 6–12 carbons) and long-chain fatty acids (LCFAs; more than 12 carbons). The majority of SCFAs in the host are derived from the microbial fermentation of indigestible carbohydrates, while MCFAs and LCFAs are mainly derived from dietary fat [4]. SCFAs are microbial bioactive metabolites which are mainly composed of acetic (C2:0), propionic (C3:0) and butyric acid (C4:0). SCFAs act as a mediator of the link between the diet and metabolism, and their anti-obesity properties are well studied [4]. Particularly, butyric acid exerts potent effects on metabolism. For example, butyric acid prevents diet-induced obesity and insulin resistance through promoting the release of the gut hormone glucagon-like peptide 1 (GLP-1) [5]. On the other hand, MCFAs, including hexanoic acid (C6:0), are found abundantly in milk and coconut oil and can be readily absorbed into the liver, where MCFAs are metabolized and used as fuel. Owing to their lipid properties, MCFAs have attracted much attention as an appropriate dietary oil for individuals with obesity and high energy demands [6]. Accumulating evidence has demonstrated that MCFAs, especially octanoic acid (C8:0) and decanoic acid (C10:0), play an important role in maintaining glucose homeostasis and lipid metabolism [7]. In human studies, patients fed an MCT-rich diet show enhanced oxygen consumption and thermogenesis, indicating that MCFAs are potent modulators of lipid and glucose metabolisms [8]. However, the effects of hexanoic acid on metabolisms have largely remained elusive.

Hexanoic acid is a free fatty acid with a six-carbon chain length and is commonly classified as an MCFA but occasionally as an SCFA. This is because hexanoic acid can be taken from foods or produced by some anaerobic bacteria [9]. In addition, hexanoic acid has been reported to activate the SCFA receptor GPR43, thereby showing unique properties [10]. Only a few studies have investigated the effect of hexanoic acid on lipid and glucose metabolisms. In the human hepatoma HepG2 cell line, hexanoic acid inhibits the increased expression of fatty acid synthase (Fasn) under stimulation with insulin or triiodothyronine [11]. On the other hand, hexanoic acid does not attenuate HFD-induced metabolic risk factors such as hyperglycemia and hyperlipidemia in LDL receptor knockout Leiden mice, which are the models of atherosclerosis in translational research [12]. Therefore, the effects of hexanoic acid on metabolisms at the whole-body level remain largely unknown. This study aims to investigate the effect of hexanoic acid on lipid and glucose metabolisms using an HFD-induced obese mouse model. An understanding of the physiological roles of hexanoic acid will help us to develop better therapeutic approaches against obesity and obesity-related diseases.

## 2. Materials and Methods

### 2.1. Animal Experiments

Male C57BL/6J mice were purchased from Japan SLC, Inc. (Shizuoka, Japan) and housed at a temperature of 24 °C and 50% relative humidity under a 12 h light/dark cycle. The 6-week-old male mice were acclimated to the CLEA Rodent Diet (CE-2; CLEA Japan, Inc., Tokyo, Japan) for 1 week prior to the dietary interventions. After acclimation to the laboratory conditions, the 7-week-old male mice were randomly divided into 4 groups (*n* = 10 in each group) and placed on the following diets for 4 weeks: a normal chow diet (ND; CE-2), a high-fat diet (HFD; D12492: 60% calories from fat; Research Diets, New Brunswick, NJ, USA), an HFD containing 5% butyric acid (HFD_C4; sodium butyrate; C4:0, FUJIFILM Wako, Osaka, Tokyo, Japan) and an HFD containing 5% hexanoic acid (HFD_C6; sodium hexanoate; C6:0, Tokyo Kasei, Itabashi, Japan). Justification of the sample size was based on a previous report [13], and sample randomization was applied using a simple random sample. The animal experiments were performed using mice adjusted for age, sex, strain and initial body weight. Mice which were obviously injured due to fighting were excluded from this study. Mice were sacrificed under deep isoflurane-induced anesthesia, and tissue samples were collected at 5 h after starvation. Blood was collected from the inferior vena cava using heparinized injections, and plasma was separated through immediate centrifugation (7000× *g*, 5 min, 4 °C). For the measurement of body weight and food consumption, two male C57BL/6J mice were housed in a cage and provided with sufficient food. Their body weight was measured once a week, and their ad libitum food intake was assessed by calculating the average of their daily food intake (g/day per mouse) for 4 weeks. The compositions of the diets are shown in Supplementary Table S1. All experimental procedures were performed in accordance with the guidelines of the Committee in the Ethics of Animal Experiments of Kyoto University Animal Experimental Committee (Lif-K24002). All efforts were made to minimize suffering.

### 2.2. Quantification of Butyric Acid and Hexanoic Acid

Butyric acid and hexanoic acid in the plasma or the liver were determined according to a previous method [13,14]. For butyric acid quantification, the liver was homogenized in distilled water (1:10, *w*/*v*), and the supernatants were collected after centrifugation (8000× *g* at 4 °C for 5 min). 5-sulfosalicylic acid dihydrate was added to the plasma samples and the liver supernatants to eliminate proteins. After centrifugation (15,000× *g* at 4 °C for 15 min), supernatants of the plasma and the liver containing an internal control (2-ethyl butyrate) were mixed with diethyl ether and centrifuged at 3000× *g* for 5 min. Butyric-acid-containing ether layers were collected and subjected to gas chromatography–mass spectrometry using a GCMS-QP2010 Ultra system (Shimadzu, Kyoto, Japan). For hexanoic acid quantification, plasma and liver samples containing the internal control (C19:0) were homogenized in methanol, followed by mixing with chloroform and water for lipid extraction. After centrifugation (2000× *g* at 17 °C for 10 min), the supernatants were collected and dried. Samples resuspended in chloroform:methanol (1:3, *v*/*v*) were subjected to liquid chromatography with tandem mass spectrometry (LC-MS/MS) using an ultra-performance LC system (UPLC, Waters, Milford, MA, USA) equipped with an Acquity UPLC system coupled to a Waters Xevo TQD mass spectrometer (Waters). The Acquity UPLC system fulfills the requirements for separation with a high resolution and sensitivity and can be used for the analysis of natural compounds. Using a methanol gradient in 10 mM ammonium formate aqueous solution, the samples were separated on an Acquity UPLC BEH C18 column (2.1 × 150 mm, 1.7 μm; Waters). Quantification of butyric acid and hexanoic acid was performed using calibration curves.

### 2.3. Biochemical Analyses

Blood glucose levels were measured using One Touch Ultra test strips (OneTouch^®^ Ultra^®^; LifeScan, Malvern, PA, USA). Plasma total cholesterol (LabAssay™ Cholesterol; FUJIFILM Wako), non-esterified fatty acid (NEFA) (LabAssay™ NEFA; FUJIFILM Wako) and insulin (Insulin enzyme-linked immunosorbent assay (ELISA) kit (RTU); Shibayagi, Shibukawa, Japan) levels were measured according to the manufacturer’s instructions. The hepatic triglyceride content was measured as previously described [13]. Briefly, mouse liver was homogenized in a mixture of chloroform/methanol/0.45 M acetic acid, and the homogenate was rotated overnight at 4 °C. After centrifugation at 1500× *g* for 10 min, the organic layer was collected, dried and resuspended in isopropanol. Plasma and hepatic triglyceride (LabAssay™ Triglyceride; FUJIFILM Wako) levels were measured according to the manufacturer’s instructions. Plasma GLP-1 (glucagon-like peptide-1 (Active) ELISA kit; Merck Millipore, Burlington, MA, USA) levels were measured using samples treated with a dipeptidyl peptidase-IV inhibitor (Merck Millipore) according to the manufacturer’s protocols.

### 2.4. Quantitative RT-PCR

Total RNA was isolated using an RNAiso Plus reagent (TAKARA, Shinjuku, Japan). Reverse transcription was performed using Moloney murine leukemia virus reverse transcriptase (Invitrogen, Carlsbad, CA, USA). cDNA was subjected to a quantitative PCR analysis using the StepOne real-time PCR system (Applied Biosystems, Waltham, MA, USA) with SYBR Premix Ex Taq II (TAKARA). Each value was normalized to *18S* rRNA and calculated using the 2-ΔΔCt method. PCR amplification was carried out using the primers described previously [13], and the primer sequences are shown in Supplementary Table S2.

### 2.5. The Intraperitoneal Glucose Tolerance Test (IPGTT) and the Insulin Tolerance Test (ITT)

Seven-week-old C57BL/6J male mice were acclimated to the CLEA Rodent Diet (CE-2; CLEA Japan, Inc.) for 1 week prior to the IPGTT or the ITT. For the intraperitoneal glucose tolerance test, mice fasted for 16 h were administered hexanoic acid (sodium hexanoate; C6:0, 2.5 g/kg body weight, Tokyo Kasei) in 0.5% carboxymethylcellulose (CMC) via oral gavage. After 1 h, the mice were given glucose (1 g/kg body weight) through an intraperitoneal injection. For the insulin tolerance test, mice fasted for 3 h were administered hexanoic acid 1 h before an intraperitoneal injection of insulin (0.75 mU/g; Sigma, Kanagawa, Japan). In both tests, blood glucose levels were monitored at 0, 15, 30, 60, 90 and 120 min after the glucose or insulin injection, and 0.5% CMC was used as control.

### 2.6. Statistical Analysis

All data are presented as the mean ± SEM. The statistical analysis was performed using GraphPad Prism 9 (GraphPad Software Inc., Boston, MA, USA). The Shapiro–Wilk test was used for an assessment of the data normality. The statistical significance of the differences between two groups was assessed using a two-tailed unpaired Student’s *t*-test, whereas that of differences between multiple groups was assessed using a one-way ANOVA followed by the Tukey–Kramer post hoc test or Dunn’s post hoc test, depending on the data normality. Statistical significance was set at *p*  <  0.05.

## 3. Results

### 3.1. Oral Administration of Butyric Acid or Hexanoic Acid Prevents HFD-Induced Obesity

**Figure 1:**
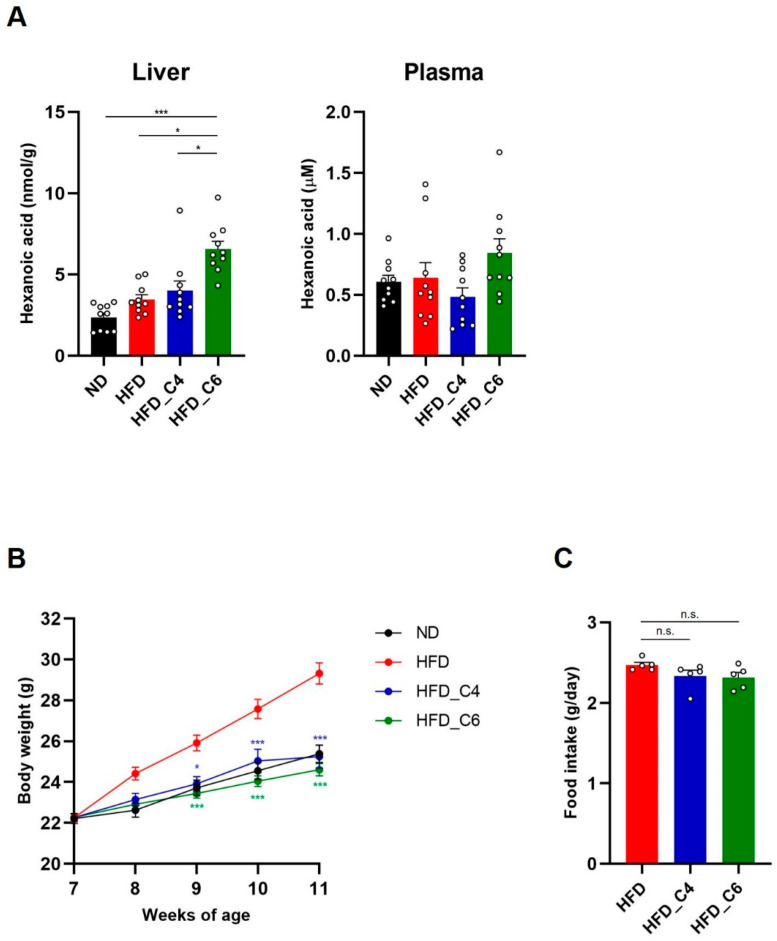
Oral supplementation of butyric acid or hexanoic acid improves HFD-induced obesity. (**A**) The liver and plasma content of hexanoic acid in mice fed a normal chow diet (ND), a high-fat diet (HFD), an HFD containing butyric acid (HFD_C4) or an HFD containing hexanoic acid (HFD_C6) (ND-fed group, *n* = 10; HFD-fed group, *n* = 10; HFD_C4 group, *n* = 10; HFD_C6 group, *n* = 10). (**B**) Body weight changes in mice fed ND, HFD, HFD_C4 and HFD_C6 (ND-fed group, *n* = 10; HFD-fed group, *n* = 10; HFD_C4 group, *n* = 10; HFD_C6 group, *n* = 10). (**C**) Food intake in mice fed HFD, HFD_C4 and HFD_C6 (HFD-fed group, *n* = 5; HFD_C4 group, *n* = 5; HFD_C6 group, *n* = 5). All data are presented as the mean ± standard error of mean (SEM). *** *p* < 0.001, * *p* < 0.05, n.s.; not significant, using a one-way ANOVA followed by Dunn’s test (**A**) or a one-way ANOVA with the post hoc Student’s *t*-test, compared to the HFD-fed group (**B**,**C**).

According to our previous study [13], male, but not female, C57BL/6J mice were fed a diet with or without FFAs, and hepatic and plasma levels of hexanoic acid were measured. Feeding with the HFD_C6 diet significantly increased the hepatic levels of hexanoic acid but not its plasma levels, indicating that hexanoic acid was rapidly absorbed into the liver via oral intake (Figure 1A). To examine whether hexanoic acid affected HFD-induced obesity, we measured body weight. After 4 weeks of feeding, the body weights of the mice fed the butyric-acid- or hexanoic-acid-containing HFD were markedly decreased compared to those of the mice fed the HFD (Figure 1B). We next measured the amount of food intake among the groups fed the HFD with or without FFAs because both FFAs possess an unpleasant flavor and odor. As a result, supplementation with butyric acid or hexanoic acid did not affect food consumption (Figure 1C). Therefore, these results indicate that both FFAs suppress HFD-induced body weight gain without reducing food consumption.

### 3.2. Butyric Acid and Hexanoic Acid Decrease Fat Accumulation and Hepatic TG Contents in Mice Fed an HFD

**Figure 2:**
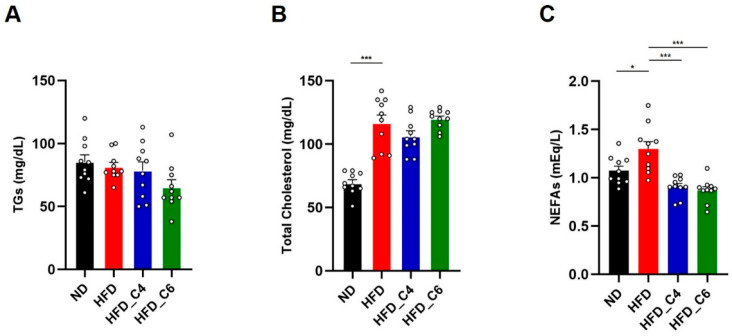
Biochemical analyses in mice fed the HFD with or without butyric acid or hexanoic acid. (**A**) Plasma triglycerides (TGs), (**B**) plasma total cholesterol and (**C**) plasma non-esterified fatty acids (NEFAs) (ND-fed group, *n* = 10; HFD-fed group, *n* = 10; HFD_C4 group, *n* = 10; HFD_C6 group, *n* = 10). All data are presented as the mean ± SEM. *** *p* < 0.001, * *p* < 0.05, one-way ANOVA followed by the Tukey–Kramer test.

**Figure 3:**
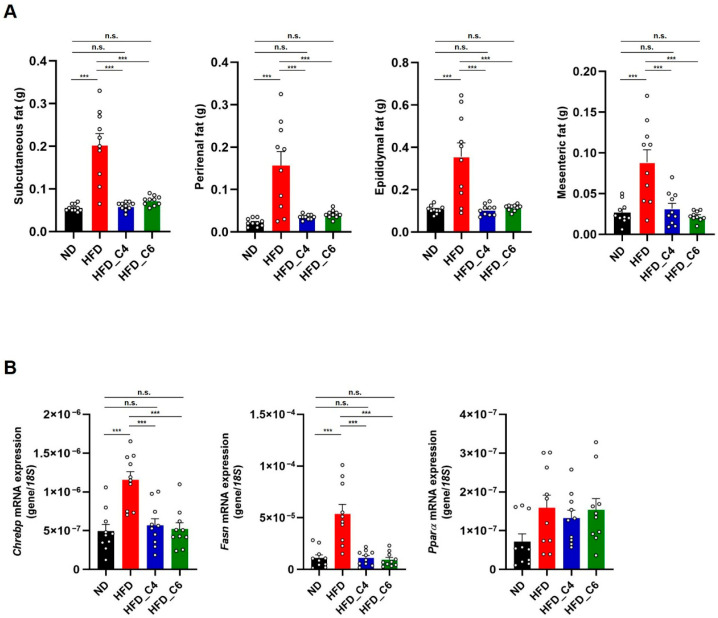
Butyric acid and hexanoic acid suppress lipid accumulation in adipose tissues under HFD feeding. (**A**) White adipose tissue weights in mice fed the ND, HFD, HFD_C4 or HFD_C6 (ND-fed group, *n* = 10; HFD-fed group, *n* = 10; HFD_C4 group, *n* = 10; HFD_C6 group, *n* = 10). (**B**) The mRNA expression levels of genes involved in lipogenesis and fatty acid oxidation in epididymal adipose tissues. *Chrebp*: MLX interacting protein-like; *Fasn*: fatty acid synthase; *PPARa*: peroxisome proliferator-activated receptor alpha. All data are presented as the mean ±SEM. *** *p* < 0.001, n.s.; not significant, a one-way ANOVA followed by the Tukey–Kramer test.

We next performed the biochemical analyses to examine the effects of each diet on plasma lipids. In accordance with the previous report, the plasma levels of triglycerides (TGs) were not changed among all groups (Figure 2A) [13]. Also, oral supplementation with butyric acid or hexanoic acid did not change the elevated plasma levels of total cholesterol (Figure 2B). However, the increased plasma levels of non-esterified fatty acids (NEFAs) under HFD feeding conditions were decreased by oral intake of butyric acid or hexanoic acid (Figure 2C). Obesity is primarily characterized by the excessive growth in the mass of the adipose tissue. Even though mice were fed an HFD for only 4 weeks, the HFD dramatically increased the mass of the white adipose tissue (subcutaneous, perirenal, epididymal and mesenteric adipose) (Figure 3A). Notably, the HFD-induced fat mass gain was suppressed in the mice fed the HFD containing either butyric acid or hexanoic acid to the same level as that in the control mice fed the ND (Figure 3A). Therefore, these results indicate that butyric acid and hexanoic acid possess potent anti-obesity properties by preventing the accumulation of fat in the white adipose tissues. We then examined the mRNA expression levels of genes involved in lipogenesis and fatty acid oxidation in the epididymal white adipose tissue. The mRNA expression levels of *MLX interacting protein-like* (*Chrebp*) and *fatty acid synthase* (*Fasn*), which play an important role in fatty acid biosynthesis, were up-regulated in the mice fed the HFD (Figure 3B). The increased mRNA expression of *Chrebp* and *Fasn* under HFD conditions was suppressed by oral intake of butyric acid or hexanoic acid (Figure 3B). In contrast, oral administration of butyric acid or hexanoic acid did not change the HFD-induced increase in *peroxisome proliferator-activated receptor alpha* (*PPARα*) mRNA expression, which is involved in fatty acid oxidation (Figure 3B).

**Figure 4:**
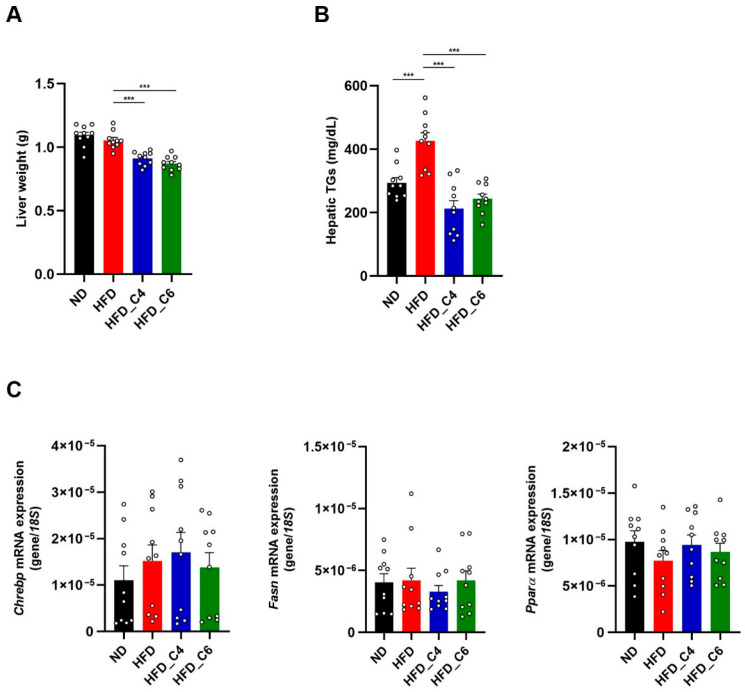
Butyric acid and hexanoic acid reduce the hepatic TG contents under HFD feeding. (**A**) Liver tissue weights and (**B**) hepatic TG contents in mice fed the ND, HFD, HFD_C4 or HFD_C6 (ND-fed group, *n* = 10; HFD-fed group, *n* = 10; HFD_C4 group, *n* = 10; HFD_C6 group, *n* = 10). (**C**) The mRNA expression levels of genes involved in lipogenesis and fatty acid oxidation in the liver. All data are presented as the mean ± SEM. *** *p* < 0.001, one-way ANOVA followed by the Tukey–Kramer test or Dunn’s test.

We next investigated the role of butyric acid and hexanoic acid in hepatic lipid metabolisms. Both FFAs effectively decreased the liver’s weight and markedly reduced the higher content of hepatic TGs in the mice fed the HFD (Figure 4A,B). However, the mRNA expression levels of *Chrebp*, *Fasn* and *PPARα* were not changed among each group (Figure 4C).

### 3.3. Hexanoic Acid Improves Glucose Metabolism Under HFD Feeding

**Figure 5:**
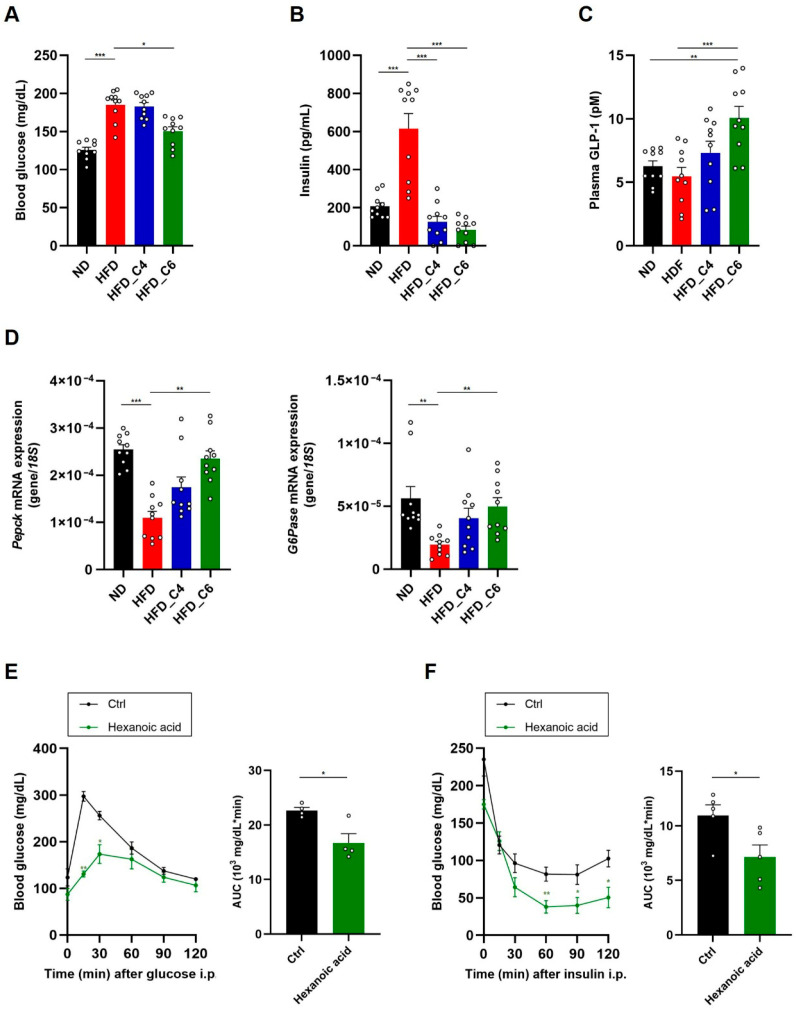
Hexanoic acid ameliorates hyperglycemia and hyperinsulinemia in mice fed an HFD. (**A**) Blood glucose, (**B**) plasma insulin and (**C**) plasma GLP-1 levels in mice fed an ND, HFD, HFD_C4 or HFD_C6 (ND-fed group, *n* = 10; HFD-fed group, *n* = 10; HFD_C4 group, *n* = 10; HFD_C6 group, *n* = 10). (**D**) The mRNA expression levels of genes involved in gluconeogenesis in the liver. *Pepck*: phosphoenolpyruvate carboxykinase 1; *G6Pase*: glucose-6-phosphatase. (**E**) The glucose tolerance test (*n* = 4) and (**F**) the insulin tolerance test (*n* = 5). All data are presented as the mean ± SEM. *** *p* < 0.001; ** *p* < 0.01; * *p* < 0.05, a one-way ANOVA followed by the Tukey–Kramer test (**A**–**C**), Dunn’s test (**D**) or a post hoc Student’s *t*-test compared to the CMC-treated group (**E**,**F**).

To investigate the effect of hexanoic acid on glucose metabolism, blood glucose levels were measured. We found that hexanoic acid, but not butyric acid, significantly improved hyperglycemia under HFD feeding (Figure 5A). Although butyric acid did not affect HFD-induced hyperglycemia, both FFAs improved HFD-induced hyperinsulinemia (Figure 5B). GLP-1 is an incretin hormone which plays an important role in the promotion of insulin secretion from the pancreatic β cells. GLP-1 receptor agonists are currently approved for use in the treatment of diabetes. We therefore investigated whether hexanoic acid affected plasma GLP-1 levels. Notably, dietary supplementation with hexanoic acid significantly increased plasma GLP-1 levels, whereas that with butyric acid failed to cause the same increase (Figure 5C). We next examined the mRNA expression levels of *phosphoenolpyruvate carboxykinase 1* (*Pepck*) and *glucose-6-phosphatase* (*G6Pase*), which are associated with gluconeogenesis in the liver, and found decreased expression of both genes in mice fed the HFD (Figure 5D). Interestingly, the HFD-induced decreased expression of both genes was restored by intake of hexanoic acid, indicating that hexanoic acid is potent in maintaining glucose homeostasis (Figure 5D). Finally, we performed the intraperitoneal glucose tolerance test (IPGTT) and the insulin tolerance test (ITT), which revealed that oral administration of hexanoic acid significantly enhanced glucose tolerance and insulin sensitivity (Figure 5E,F). Taken together, these results suggest that hexanoic acid is a potent FFA which improves hyperglycemia under HFD feeding by enhancing insulin sensitivity.

## 4. Discussion

In this study, we investigated the effect of hexanoic acid on lipid and glucose metabolisms under HFD conditions. We showed the anti-obesity efficacy of hexanoic acid here, which was comparable to that of butyric acid. Accumulating evidence has revealed that butyric acid prevents diet-induced obesity and insulin resistance. Oral supplementation with butyric acid enhances fatty acid oxidation and energy expenditure through up-regulating the expression of peroxisome proliferator-activated receptor gamma coactivator-1a and uncoupling protein 1 in the brown adipose tissue [15]. One mechanism through which butyric acid improves lipid and glucose metabolisms is through activation of the SCFA receptors. The role of the SCFA receptors in energy metabolism has been well studied, especially for GPR41 and GPR43. In our previous report, we demonstrated that SCFAs, including butyric acid, conferred metabolic benefits for mice fed an HFD, but these beneficial effects of SCFAs were abolished in *Gpr41* gene knockout mice [13]. Additionally, we showed that SCFAs enhanced the release of noradrenaline from the sympathetic nerves and promoted energy expenditure through GPR41 activation [4]. Therefore, SCFAs, including butyric acid, exert favorable effects on metabolic health, in part through activating GPR41. Also, GPR43 plays an important role in energy metabolism. We previously reported that *Gpr43* gene knockout mice exhibited a decreased β cell mass and developed glucose intolerance due to impaired insulin secretion under HFD feeding [4]. Furthermore, *Gpr43* deletion mice showed obesity, whereas mice overexpressing GPR43 only in the adipose tissue were lean under normal diet conditions [4]. We also found that the activation of GPR43 suppressed adipose insulin signaling and increased the expressions of genes related to energy expenditure, glycolysis and β-oxidation in the muscles. Therefore, GPR43 prevents excessive fat accumulation in the white adipose tissues and promotes the utilization of unincorporated lipids and glucose in the muscles. Together, these studies suggest that GPR43 as well as GPR41 play an essential role in the regulation of host metabolisms. However, SCFA supplementation improves metabolic functions in *Gpr43* knockout mice fed an HFD [13], raising the possibility that GPR41 is more likely to be involved in this improvement in metabolism when SCFAs are orally administrated.

In the present study, we revealed that hexanoic acid improved obesity and lipid metabolism in mice fed an HFD to the same extent as butyric acid. This thus indicates the potent efficacy of hexanoic acid in the treatment of obesity and obesity-related diseases. In contrast to LCFAs, which are incorporated into the chylomicrons as a triglyceride, dietary MCFAs are directly transported to the liver and rapidly metabolized by mitochondrial β-oxidation. Therefore, dietary MCFAs affect hepatic lipid metabolisms. Indeed, octanoic acid (C8:0) and decanoic acid (C10:0) reduce hepatic lipid accumulation through decreased lipogenesis and increased lipolysis [16]. In the present study, feeding with hexanoic acid significantly increased hepatic levels of hexanoic acid. Although no significant changes were observed, the plasma levels of TGs tended to decrease in the mice fed HFD_C6. Therefore, hexanoic acid was rapidly absorbed and directly transported to the liver via oral intake, resulting in a reduction in plasma TG contents. Furthermore, hexanoic acid suppressed the increased expression of *Chrebp* and *Fasn* under HFD feeding but not that of PPARα in the adipose tissues, suggesting that hexanoic acid exerts its effects in part by inhibiting lipogenesis rather than by promoting fatty acid oxidation. Although the underlying mechanisms linking hexanoic acid to a metabolic benefit remain unclear, our results suggest that hexanoic acid, as well as butyric acid, is a potent FFA with anti-obesity properties.

Butyric acid also plays an important role in the regulation of glucose metabolism and insulin sensitivity. It is well known that the elevated hepatic lipid deposition under HFD feeding causes insulin resistance. Butyric acid has been reported to offer protection from HFD-induced hyperinsulinemia and improve glucose tolerance [13,17]. Also, butyric acid promotes incretin secretion and gluconeogenesis in the intestines via the gut–brain axis [4,18]. Equally, supplementation with inulin-type fructan, which promotes the growth and activity of butyric-acid-producing bacteria, improves glucose intolerance [19]. Consistent with our previous report [13], we showed here that butyric acid improved hyperinsulinemia in mice fed an HFD but failed to suppress hyperglycemia. In contrast, hexanoic acid improved both hyperinsulinemia and hyperglycemia and increased insulin sensitivity and plasma GLP-1 levels. GLP-1 is a gut hormone with anti-diabetes and anti-obesity effects, and agonists of the GLP-1 receptor are used for the treatment of type 2 diabetes and obesity. Therefore, these results suggest that hexanoic acid is a potential candidate functional food ingredient in the prevention of type 2 diabetes and obesity. Collectively, our findings shed light on the positive effects of hexanoic acid on lipid and glucose metabolisms at the whole-body level.

To our knowledge, the receptor responsible for hexanoic acid has not been well defined.

Although GPR43 was reported to be activated by hexanoic acid, the physical abundance of hexanoic acid under normal diet conditions is not enough to activate GPR43. Other than GPR43, the MCFA receptor GPR84 is a plausible receptor for hexanoic acid. We previously reported that the oral administration of medium-chain triglycerides (MCTs) enhanced glucose tolerance, and capric acid increased GLP-1 secretion by activating GPR84 in enteroendocrine STC-1 cells [7]. Furthermore, we demonstrated that activation of GPR84 by capric or lauric acid protected the liver from diet-induced lipotoxicity [14]. Therefore, these studies indicate that GPR84 plays an important role in the regulation of metabolisms. However, hexanoic acid was not identified as a ligand for GPR84 by our screening [14], and thus, further investigations are needed to clarify the molecular mechanisms of hexanoic action in the regulation of metabolisms.

SCFAs are the major end products of microbial fermentation in both ruminants and non-ruminants, and their amounts and tissue components differ markedly among species. In ruminant animals, SCFAs are produced from microorganisms in the forestomach and are rapidly absorbed, which provide nearly 80% of the energy requirement of the animal. In mammals, including humans, the majority of the SCFAs produced in the colon are utilized by the epithelial colonic cells as fuel, and thus, they provide only 10% of the energy required by the cells in the whole body [20]. Therefore, a higher amount of SCFAs and hexanoic acid is present in the peripheral circulation of ruminant animals than that in mammals. The level of circulating FFAs affects milk fat composition. Cow milk, for example, contains hexanoic acid, but human breast milk does not. This indicates that dietary intake of hexanoic acid is required for us to improve metabolic health. In addition to this, the promotion of endogenous SCFA production is also useful for improving metabolic functions. Dietary fiber is used as a substrate for anaerobic fermentation by the gut microbiota, which produces SCFAs and reduces the risk of obesity and type 2 diabetes. In human patients with obesity-related disorders, the composition of the gut microbiota is altered (dysbiosis). Microbial dysbiosis in the gut is tightly associated with metabolic disorders, and hence, the use of prebiotics such as dietary fibers and probiotics such as butyrate-producing bacteria has received much attention for preventing and treating obesity and obesity-related diseases in recent years. Although we showed here that hexanoic acid improves metabolic health under HFD conditions, the effect of hexanoic acid on the gut’s microbial composition remains unclear. HFD feeding is a well-known feeding method that causes dysbiosis and exacerbates lipid and glucose metabolisms by altering the production of microbial metabolites such as SCFAs. Recently, a novel probiotic strain, *Ligilactobacillus animalis* (*L. animalis*) LA-1, was identified [21]. Oral administration of *L. animalis* LA-1 inhibits HFD-induced weight gain, hepatic lipid accumulation and adipose tissue hypertrophy by restoring the gut’s microbial composition. Restoration of the gut microbiota following *animalis* LA-1 treatment results in an increase in total SCFA levels. Therefore, maintenance of gut microbial homeostasis is important for metabolic health. Future studies are needed to address how and whether hexanoic acid affects the gut microbiota under HFD feeding.

Obesity and obesity-related diseases are now worldwide health problems. To solve these problems, effective therapeutic strategies such as developing drugs and supplements are required. Although this study revealed that hexanoic acid is beneficial for improving metabolic health, its effects on human metabolisms remain elusive. A recent study examining the effect of hexanoic acid on human metabolisms has demonstrated that oral administration of Akovita SCT, an easily consumable butyric- and hexanoic-acid-enriched sunflower oil, increases circulating levels of both FFAs but does not change metabolic parameters (plasma levels of glucose, insulin and triglycerides) in men with obesity [22]. In this human trial, the participants received SCFAs (ranging from 650 to 2000 mg with a 2:1 ratio for butyric acid:hexanoic acid), and their metabolic parameters were examined 6 h after ingestion. Therefore, the dose and duration of hexanoic acid administration in this human study are quite different from those in our mouse study. Further investigations are needed to evaluate the effects of hexanoic acid on human metabolic health. Equally, the molecular mechanisms behind the anti-obesity and anti-diabetic effects of hexanoic acid also remain elusive. However, considering that hexanoic acid is a remarkable FFA with anti-obesity and anti-diabetes properties, hexanoic acid is a promising candidate for the treatment of obesity-related disorders such as type 2 diabetes and cardiovascular diseases. Taken together, our results help enrich our understanding of the role of hexanoic acid in improving metabolic function.

## 5. Conclusions

We here demonstrated that hexanoic acid is beneficial for improving metabolic health under HFD-feeding. Hexanoic acid suppresses HFD-induced obesity and fat accumulation in white adipose tissues in part by inhibiting lipogenesis rather than by promoting fatty acid oxidation. Furthermore, hexanoic acid improves hyperinsulinemia and hyperglycemia under HFD condition, and enhances glucose tolerance and insulin sensitivity. Notably, the anti-obesity efficacy of hexanoic acid is comparable to that of butyric acid which is well-known FFA with anti-obesity property, and the anti-diabetes property of hexanoic acid is more potent than that of butyric acid. For practical applications, human studies are needed to evaluate the effects of hexanoic acid on human metabolic health. Further investigation will provide insight into the molecular mechanisms underlying the beneficial effect of hexanoic acid on maintaining lipid and glucose metabolisms. Taken together, our findings suggest that hexanoic acid is a promising candidate as functional food ingredients in the prevention of obesity and type 2 diabetes.

## Data Availability

All data are included in this article and its Supplementary Materials and are available from the corresponding author upon reasonable request.

## Supplementary Materials

The following supporting information can be downloaded at https://www.mdpi.com/article/10.3390/nu17172868/s1. Table S1. Dietary composition of high-fat diet used in this study; Table S2. Primer sequences used in this study.
