# Supplementary material for: Hexanoic Acid Improves Metabolic Health in Mice Fed High-Fat Diet
Source: Nutrients. 2025 Sep 4;17(17):2868. doi: 10.3390/nu17172868 (PMC12430857; doi:10.3390/nu17172868)
Supplement: Supplementary file 1 [file nutrients-17-02868-s001.zip › nutrients-3832854-supplementary.pdf]

Supplementary Material

Supplementary Table S1. Dietary composition of high-fat diet used in this study. HFD, high-fat diet; HFD_C4, HFD containing 5% butyric acid; HFD_C6, HFD containing 5% hexanoic acid.

Formula	HFD	HFD_C4	HFD_C6
Product		kcal %	
Protein	20	20	20
Carbohydrate	20	20	20
Fat	60	60	60
Ingredient		gm	
Casein, 30 mesh	200	190	190
L-cystine	3	2.85	2.85
Maltodextrin 10	125	118.25	118.25
Sucrose	68.8	65.36	65.36
Cellulose, BW200	50	47.5	47.5
Butyric acid	0	38.6925	0
Hexanoic acid	0	0	38.6925
Soybean oil	25	23.75	23.75
Lard	245	232.75	232.75
Mineral mix S10026	10	9.5	9.5
Dicalcium phosphate	13	12.35	12.35
Calcium carbonate	5.5	5.225	5.225
Potassium citrate. 1 H_2O	16.5	15.675	15.675
Vitamin mix V10001	10	9.5	9.5
Choline bitartrate	2	1.9	1.9
FD&C blue dye*	0.05	0.0475	0.0475

*FD&C blue dye: synthetic organic compound primarily used as a blue colorant for dietary supplements.

Supplementary Table S2. Primer sequences used in this study.

	Forward	Reverse
18s	5'-acgctgagccagtcagtgta-3'	5'-cttagagggacaagtggcg-3'
Chrebp	5'-ctggggacctaaacaggagc-3'	5'-gaagccaccctatagctccc-3'
Fasn	5'-gctgcggaaacttcaggaaat-3'	5'-agagacgtgtcactcctggactt-3'
PPAR α	5'-cctgaacatcgagtgtcgaa-3'	5'-ggccttgaccttgttcatgt-3'
Pepck	5'-ccacagctgctgcagaaca-3'	5'-gaagggtcgcatggcaaa-3'
G6Pase	5'-ccatgcaaaggactaggaacaa-3'	5'-taccagggccgatgtcaac-3'
